# Investigating Physio-Thermo-Mechanical Properties of Polyurethane and Thermoplastics Nanocomposite in Various Applications

**DOI:** 10.3390/polym13152467

**Published:** 2021-07-27

**Authors:** Tyser Allami, Ahmed Alamiery, Mohamed H. Nassir, Amir H. Kadhum

**Affiliations:** Department of Chemical and Process Engineering, Faculty of Engineering and Built Environment, Universiti Kebangsaan Malaysia, Bangi 43600, Selangor Darul Ehsan, Malaysia; dr.ahmed1975@ukm.edu.my (A.A.); mhnassir1949@gmail.com (M.H.N.); amir8@ukm.edu.my (A.H.K.)

**Keywords:** nanocomposite, physio-thermo-mechanical properties, polyurethane, polymers, bio-applications

## Abstract

The effect of the soft and hard polyurethane (PU) segments caused by the hydrogen link in phase-separation kinetics was studied to investigate the morphological annealing of PU and thermoplastic polyurethane (TPU). The significance of the segmented PUs is to achieve enough stability for further applications in biomedical and environmental fields. In addition, other research focuses on widening the plastic features and adjusting the PU–polyimide ratio to create elastomer of the poly(urethane-imide). Regarding TPU- and PU-nanocomposite, numerous studies investigated the incorporation of inorganic nanofillers such as carbon or clay to incorporating TPU-nanocomposite in several applications. Additionally, the complete exfoliation was observed up to 5% and 3% of TPU–clay modified with 12 amino lauric acid and benzidine, respectively. PU-nanocomposite of 5 wt.% Cloisite^®^30B showed an increase in modulus and tensile strength by 110% and 160%, respectively. However, the nanocomposite PU-0.5 wt.% Carbone Nanotubes (CNTs) show an increase in the tensile modulus by 30% to 90% for blown and flat films, respectively. Coating PU influences stress-strain behavior because of the interaction between the soft segment and physical crosslinkers. The thermophysical properties of the TPU matrix have shown two glass transition temperatures (*T_g_*’s) corresponding to the soft and the hard segment. Adding a small amount of tethered clay shifts *T_g_* for both segments by 44 °C and 13 °C, respectively, while adding clay from 1 to 5 wt.% results in increasing the thermal stability of TPU composite from 12 to 34 °C, respectively. The differential scanning calorimetry (DSC) was used to investigate the phase structure of PU dispersion, showing an increase in thermal stability, solubility, and flexibility. Regarding the electrical properties, the maximum piezoresistivity (10 S/m) of 7.4 wt.% MWCNT was enhanced by 92.92%. The chemical structure of the PU–CNT composite has shown a degree of agglomeration under disruption of the sp2 carbon structure. However, with extended graphene loading to 5.7 wt.%, piezoresistivity could hit 10^−1^ S/m, less than 100 times that of PU. In addition to electrical properties, the acoustic behavior of MWCNT (0.35 wt.%)/SiO_2_ (0.2 wt.%)/PU has shown sound absorption of 80 dB compared to the PU foam sample. Other nanofillers, such as SiO_2_, TiO_2_, ZnO, Al_2_O_3_, were studied showing an improvement in the thermal stability of the polymer and enhancing scratch and abrasion resistance.

## 1. Introduction

A range of research has been conducted on the ongoing discussion of the effect of chemical structure and annealing of the morphology of polyurethanes (PUs) on experimental and theoretical work on phase-separation kinetics [1]. Phase separation is the most crucial reason for the PUs’ microphase separation as the powerful hydrogen link between the urethane hard parts [2]. The main components of PUs are macrodiol, diisocyanate, and chain extender [3]. The chemistry of PU synthesis depends on the reactions of isocyanate. The hydrolysis resistance of PUs and the noting of the diol chemistry on molecular weight stability in water are discussed by Gomez et al. [4].

PUs belong to a group of elastomers that are linked to a urethane material with a distinctive feature of being hard and soft parts in the macromolecule [5]. In addition, the environmental issue that involves PU recycling was a concern of researchers [6]. The synthesis of PU is the main theme of the studies in the field of working towards environmentally based materials such as PU, using the short-chain diol called diisocyanate [7]. Polyurethane is widely considered as the biggest polymer product which is categorized under plastics [8]. Plastic and modular construction industries produce big quantities of PU wastes in the fabrication process during either processing or utilization of materials [9]. The structure and properties of PU parts have been investigated to achieve better biocompatibility and are characterized by surface and bulk morphology [10]. Traditionally, PU items are equipped with many organic solvents and free isocyanate monomers [11]. In the production of PU foams, catalysts are employed in the polyaddition reaction [12]. It has been reported that two simultaneous reactions could occur during PU manufacturing that involves the isocyanates and polyols during gas liberation or a foaming reaction [13]. As an example, paints from modified PU are extensively used as topcoats for corrosion and weather resistance. The aliphatic PU is sensitive to acrylic ester comprising hydroxyl which is widely considered as having good adhesion and being aging-resistant [14]. These processes involve building up strong hydrogen links of the PU matrix, raising the rigidity of the matrix, and negatively affecting the relaxation of dipoles [15]. The particles dispersed in aqueous phase dispersion, classified as anionic PU, are binary colloidal systems, cationic, and non-ionic systems [16]. Regarding the nanomaterials, the antibacterial polyurethane activity of composite nanofibers has been assessed in food-borne pathogenic bacteria and staphylococcus aureus, using various techniques [17]. The focus is on the formation of soft, long-chain diol segments, whereas the construction of the hard segment belongs to the microdomain of segmented PU copolymers [18].

**Figure 1:**
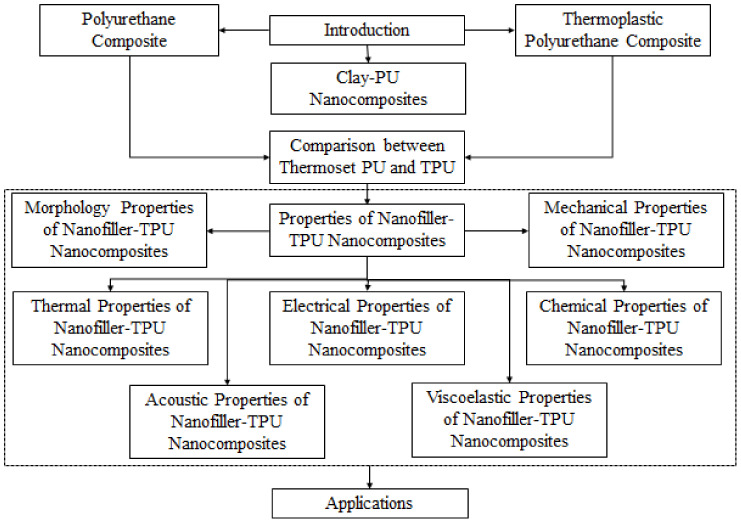
Organization of the paper.

The organization of this review paper is explained in Figure 1. The introduction is followed by shedding the light on the general properties of PU composite, TPU composite, a comparison between PU and TPU, and PU clay. The other main section of the paper is presented to discuss the properties of the nanofillers in both PU and TPU. These properties include morphology, mechanical properties, thermal properties, electrical properties, acoustic properties, and viscoelastic properties. The last main section of the paper is devoted to highlighting some applications in various fields.

## 2. Polyurethane Composite

The soft segment relative to the hard segment of the lower air-interfacial polymer has made the appearance of PU in polyol structure [19]. The size of the dispersed PU particle reduces the concentration of the ionic group per unit chain length of the PU pre-polymer [20]. On the other hand, the increase of the ratio of hard to soft parts of the PU chains results in increasing the viscosity of the prepolymer [21]. The reduction of the ionic group concentration and the increase in the viscosity could cause a stiffer PU chain, lowering the solubility of the polymer, enhance the phase separation, and make coarse particles at the surface [22]. The above behavioural changes increase the hysteresis values of the quaternized polymers compared to the base PUs. The effect then causes a noticeable rearrangement surface that changes the hydration [23].

PUs are industrially crucial polymers with a range of structures and uses [24]. The acrylics PU polymer is known by a table of Newtonian rheological features [25]. Regarding the aging of the PU film, Sanchis et al. [26] have shown that the PU structure plays an important role in addressing the age issue.

The poor elasticity of the two segmented PUs was caused by their low molecular weight which, in turn, influences the morphology of the segmented PUs [27]. Hetflejš et al. [28] illustrated that the stabilizing efficiency of polymer-linked structures could be compared with that of their low-molecular-weight analogs, physically admixed with the PU. In another field, a sufficient mixing of the fluoro acrylic and PU polymers in the film properties could produce better results [29]. In this regard, it was found that modifying the ratio of PU and polyimide parts results in widening the range of properties from plastic to elastomer of the poly(urethane-imide) [30]. The solubility of the polyimide is enhanced as a result of adding isocyanate-terminated PU prepolymer, making a gelatine solution [31].

## 3. Thermoplastic Polyurethane Composite

Thermoplastic polyurethanes (TPUs) are linear segmented copolymers composed of hard and soft segments separated by a microphase, which complicates the investigation of its relevant microstructure [32]. The difficulty of this investigation shortening the research of the microstructure to extension behaviour [33]. Thermoplastic polyurethane (TPU) and the thermoset PU are chemically similar to each other; however, they have different features [34]. TPU is characterized by unique physical-chemical properties due to the reformation ability from melting state which makes it elastic, highly flexible, and suitable for many industrial applications [35]. On the other hand, the sensitivity of TPU to oil is smaller than that of thermoset PU, as the latter is easy to tear with abrasive applications [36]. TPU becomes soft at high temperatures and can hold low pressure, possessing a higher tensile modulus in comparison to rubber [37].

TPU is generally described as “bridging the gap between rubber and plastics” and imparts high elasticity combined with high abrasion resistance, and, hence, becomes suitable to a variety of biomedical applications [38]. TPUs are conventionally not degradable; however, they become susceptible to hydrolytic and oxidative under vacuum [39]. The TPUs’ susceptibility to such degradation causes a problem for long-lasting biomedical implants which exploit designing biodegradable PUs [40].

TPU displays a very wide range of properties, ranging from very soft to strong, rigid thermoplastics that depend on the chemical compositions, backbone structures, and resultant microphase morphologies [41]. The 40-year investigation revealed that there are various morphological models for segmented PUs [42]. TPU has become one of the most versatile engineering thermoplastics that have constituted developing more interesting polymers due to specific structures of TPU macromolecules, interphase interactions, and microphase transformations [43]. Several researchers have addressed that blending TPU with nanomaterials enhances its physical properties and toughness [44]. The TPU’s good compatibility with polycarbonate or acrylonitrile butadiene styrene was behind using TPU as a modifier to create new blends [45]. The effect of using special additives can be seen in creating properties necessary to achieve flame retardance, antistatic, and radiation crosslinking ability [46]. Besides, exposing TPU to severe conditions results in significant structural changes depending on the structure and morphology; however, such changes deteriorate the physical properties [47].

## 4. Comparison between Thermoset PU and TPU

Thermoset PU and TPU belong to two different classes of polymers; however, they show some differences; for example, the thermoplastic has low melting points while the thermoplastic can withstand high temperature. TPU can be remoulded, while thermoset cannot be reformed, remoulded, or recycled. Table 1 shows the main differences between thermoset PU and TPU [48].

## 5. Clay–PU Nanocomposites

The valuable and key properties of TPU that have been collected by Tehran et al. [49] were articulated in Table 2. There were three ways to enhance these key properties: the first is via manipulating the three basic building blocks, which are polyester/polyether polyol, diisocyanates, and chain extender; secondly, blending with an appropriate polymer; and thirdly, incorporation of inorganic fillers, particularly nanofillers, into the PU matrix. Amongst these three techniques, the incorporation of inorganic nanofillers was found to be the best, in terms of both commercial effectiveness and technical viability of the process [46]. This is because nanofillers have great advantages for improving barrier and mechanical properties, as well as thermal stability [50].

It has been observed that montmorillonite clay modified with long-chain onium ions exhibited excellent compatibility with several polyols that are commonly used for PU synthesis [51]. Additionally, an increase in the chain length of alkyl groups that are present in the long-chain onium ions causes an increase in interlayer distance between the clay platelets in the nanocomposites. Clay–polymer nanocomposites may be prepared by three distinct methods, e.g., melt blending, solution mixing, and in-situ synthesis. In the case of the melt blending technique, nanoclay is mixed with the molten polymer in an internal mixer or extruder. Meanwhile, solution mixing involves the intermixing of a solution of polymer and the solvated nanoclay, utilizing mechanical stirring and/or ultrasonic vibration, followed by the evaporation of the solvent. In the case of the in-situ synthesis technique, clay is dispersed either with the polyol or with the prepolymer followed by a further course of polymerization. Several factors that are responsible for the dispersion of nanoclay [52] in the polymer matrix such as the method of preparation, mixing temperature and thermal history, mixing time, shear rate, the extent of deformation, the solvent used (particularly in solution mixing as well as in-situ preparation), the concentration of polymer solution (again in solution mixing and in-situ preparation), the molecular weight of the monomer (for the in-situ preparation method) or polymer, the types of modifier used for modification of nanoclay and the extent of modification of the nanoclay [53].

## 6. Properties of Nanofiller–TPU Nanocomposites

**Figure 2:**
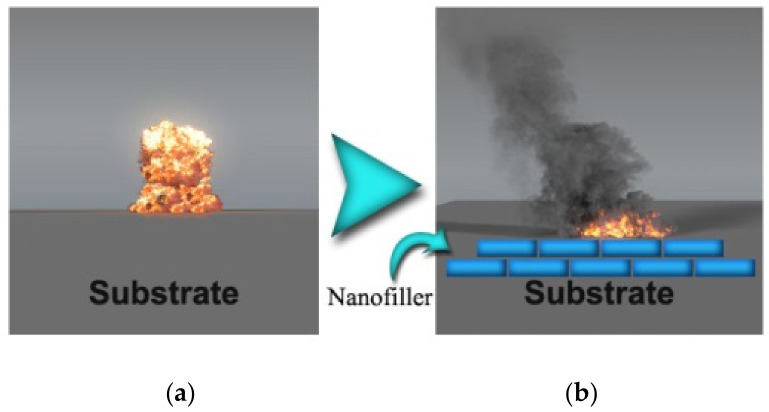
(**a**) The mechanism of nanofiller on the substrate and (**b**) corresponding morphology [54].

The main application of TPU is in the medical field due to its exceptional mechanical properties and biocompatibility. The siloxane-based TPU is one of the most important nanocomposites. TPU nanocomposites were vigorously studied based on the nanofiller aspect ratio, surface modification, and percentage loading. The mechanical properties were increased at an even small amount of loading. In this section, several TPU nanocomposites will be discussed. The mechanism of the nanofillers is explained schematically, as shown in Figure 2 [54]. The effect on nanofillers will be investigated in terms of morphology, mechanical properties, thermal properties, chemical properties, electrical properties, acoustic properties, and viscoelastic properties.

### 6.1. Morphology Properties of Nanofiller–TPU Nanocomposites

The first-ever successful attempt of dispersion of nanoclays in the TPU matrix has resulted in incredibly high interest amongst the scientific community [55]. It was seen that the morphology of clay platelets plays a crucial role in the improvement of the properties of the clay–TPU nanocomposites. The effect of hard segment content along with the amount of clay on the morphology has been discussed by Xu et al. [56]. Increased hard segment content has been seen to result in an increase of the basal spacing of the clay platelets at a lower clay content. However, the opposition has been observed to occur at higher clay contents, where increased hard segment content reduces the basal spacing. Two varieties of modified montmorillonite (MMT) have been dispersed in the TPU matrix [57]; the first is clay modified with 12 amino lauric acids and the second is clay modified with benzidine. This was done in order to study the effect of the modifier on the morphology and properties of the resulting nanocomposites [57]. Complete exfoliation was observed of up to 5% and 3% for clay modified with 12 amino lauric acid and benzidine, respectively. 

An investigation into an intercalated to exfoliated morphology was carried out, in which there was the incorporation of clay modified with dilauryldimethyl ammonium bromide and 4,4′-diaminodiphenylmethane, respectively [58]. Intercalated structures were mainly observed when the clay was used as a pseudo chain extender [59]. However, the prevalence was as follows: firstly, intercalated morphology with nontethered clay (clay that was mixed physically), and secondly, exfoliated morphology with the tethered clay (clay that had the active functional group)—where both were for nanocomposite prepared by in-situ preparation technique [60]. It has also been shown by many other researchers that tethering of the clay leads to exfoliation of the clay platelets [61]. Of note is the fact that highly exfoliated morphology occurrence up to 40 wt.% of nanoclay was demonstrated by using clay as a pseudo chain extender [62]. The effect of changing the number of end-tethered–OH functional groups on the tail of the modifier (the modification of montmorillonite) has been studied previously [63]. The morphology of the clay changes from intercalated to exfoliated, with the increase in the number of [–OH] groups in the modifier. The MMT with tris (hydroxymethyl) aminomethane was modified so that a tethered clay with three [–OH] groups may be prepared [62]. It is of importance to note that the intercalate-to-exfoliate morphology was for all the nanocomposites and increased aggregation tendency was also seen but only at higher clay contents [64]. High mechanical shearing (not the tethering of the clay) was found by Adak et al. [52] as a complete exfoliation of the nanoclay. The clay that possessed the in-house synthesized organizer was modified before subsequently being dispersed into the TPU matrix [52].

**Figure 3:**
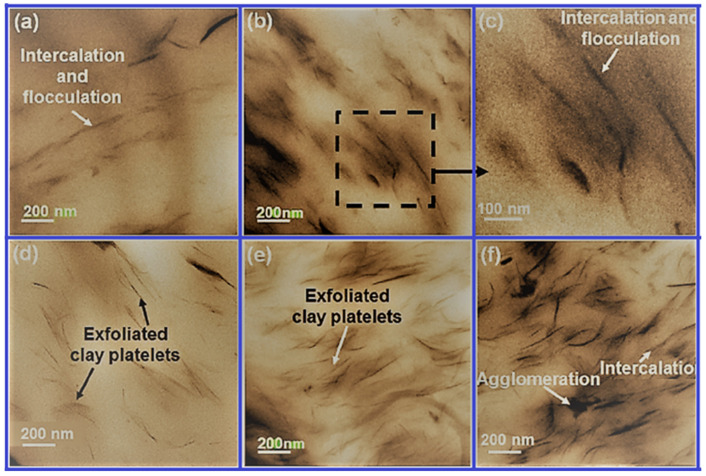
Images showing dispersion of clay-platelets in CPN: (**a**) PU-CA-1, (**b**,**c**) PU-CA-3, (**d**) PU-C30B-1, (**e**) PU-C30B-3, (**f**) PU-C30B-5. [52].

Figure 3 shows the overall impact of nanofillers on the morphology of C30B-CPN and CA-CPN, highlighting the state of dispersion of clay layers in the PU matrix. In the case of CACPN, an intercalated and flocculated morphology was obtained where the clay layers were oriented in the PU matrix by edge–edge interaction. Good dispersion was observed at lower clay concentrations. However, at higher clay-loadings, clusters or particles were formed due to agglomeration of clay-layers, leading to non-uniform dispersion in the PU matrix. Other effects can be seen in the subsequent parts of Figure 3 [52].

### 6.2. Mechanical Properties of Nanofiller–TPU Nanocomposites

The mechanical properties of modified TPU are extremely important because of the usefulness of modified TPU in many engineering applications. It has been observed that the addition of nanoclay into the PU matrix improves the tensile properties to a significant degree [65]. As an example, adding 10 wt.% of modified clay increases the tensile strength, modulus, and strain at the break by more than 100% [45,56]. Young’s modulus of the nanoclay–TPU nanocomposites has previously been seen to increase with the addition of modified nanoclays [66]. Nonetheless, improved Young’s modulus coupled with a reduction in tensile strength and elongation at break with the addition of clay has also been reported in previous studies [67]. Besides that, the destruction of hydrogen bonds in the hard segment of TPU due to the incorporation of tethered nanoclays has also been observed [67]. The destruction in H-bonding occurs due to the H-bond formation between the carbonyl group of the TPU and the [–OH] group present on the tail of the modifier to the clay. However, modulus and tensile strength are increased by 110 and 160%, respectively, for the nanocomposite containing 5 wt.% of Cloisite^®^30B. The reaction of the surface [–OH] group of the MMT with the isocyanate during in situ synthesis, which leads to an improvement in tensile strength, as well as elongation at break, has been observed from experimentation [56].

The effect of the modifier on the improvement in the properties of the TPU has been studied using three types of modifier [68]. With the increase in the degree of exfoliation, the tensile strength and elongation at break have been found to increase. However, increasing the mixing time (in order to achieve exfoliation) is found to cause degradation in the TPU matrix [69]. It is reported that better properties are obtained when the degree of exfoliation of the clay platelets is higher. This is due to an increase in the mass-to-volume interaction between the clay and polymer in the exfoliated state of clay in the polymer matrix, which in turn is highly dependent on the type of modifier used. The samples with 0.5 wt.% CNTs, an increase of the tensile modulus concerning the TPU matrix was estimated between 90 to 30% for flat and blown films, respectively. It is also shown that the samples of CNT–TPU nanocomposites show that the stiffness and tensile strength increase with increasing content of CNTs at the expense of elongation at break which is reduced by more than 40%. The typical stress–strain curves for the sample films containing 0, 0.2, 0.5, and 1 wt.% multiwall carbon nanotubes (MWNTs). Results show a non-monotonic trend of mechanical properties with the filler loading [70].

It has been previously reported that the rate and degree of separation have a direct correlation with the tensile features of PU elastomers [71]. The significant finding of this current work was depicting the role diisocyanate symmetry played in the stage of development of microphase separated morphology and the resultant new mechanical properties of the PU [5]. The method deemed to be best to improve the PUs’ mechanical properties is the chemical linking of the PU chain with functionalized dendritic polymers through crosslinking [72]. Energy recovery and mechanical, chemical, and thermochemical recycling were some of the ways identified for recycling PU [73]. The findings may be linked to the high porosity, as well as the weak compressive toughness of alveolar PU. The high decrease in the strength was because of the rise in PU foam content which in turn was a result of an increase in mass loss [74]. Moreover, the static tensile features and toughness of the PUs are depicted in Table 3 [75].

**Figure 4:**
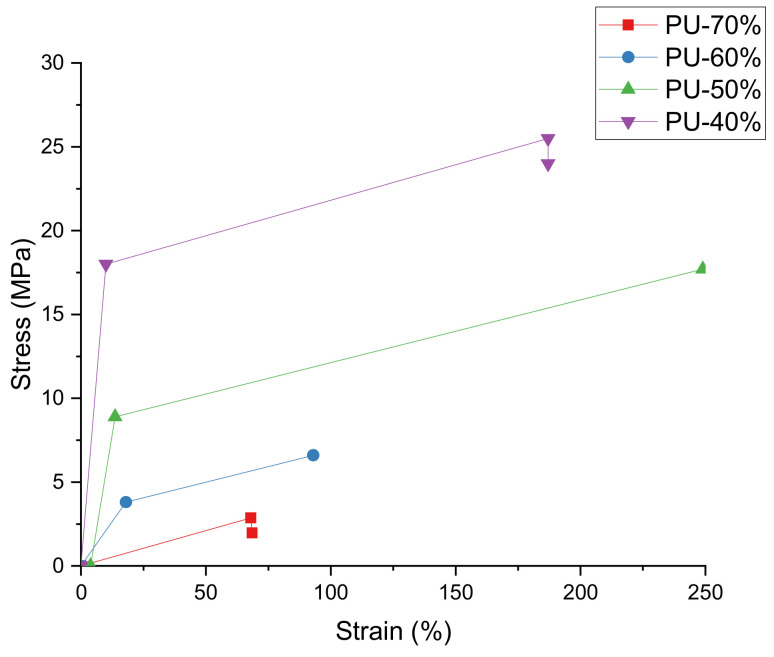
Stress–strain behavior of segmented PUs [84].

Linear poly(urethane-imide) elastomers were achieved [76]. They showed significantly better mechanical features and greater thermal stability when compared to the typical linear PU [76]. The patterns were the same as the flexible PU but accompanied by a reduction in stiffness and strength in the wet condition where new samples showed enhanced features for all aging states [77]. By catalytically balancing two deeply understood reaction schemes, flexible PU foams are made. Flexible PU foams are produced by balancing two well-understood reaction schemes through catalytic balancing. Besides that, an analysis of the orientation-elongation feature of linear PU elastomers was shown as having low deformation with the hard segment’s base transversing to the stretching direction [78]. As a pigment grinding medium, the phosphate PU acrylics were shown to be well-suited for this function because of their sheer stability [79]. It has been stated that there may be improved cracking when steroids were adsorbed on PUs [80]. Looking at drying time, PU resin-coated panels were tested for four different properties, which were scratch hardness, impact hardness, flexibility, and chemical barrier [81]. It should be noted that for PUs comprising both hydrophobic fluorinated chain as well as hydrophilic phosphatidylcholine group, the need existed for the utilization of the average tapping mode to examine the difference of surface stiffness between hard and soft PU segments [82]. Arrieta et al. [83] maintained that higher modulus was seen compared to DCE-based PUs, but equal tensile strength was also present due to higher Mn content [83]. Xu et al. [84] observed that the rubbery plateau modulus segmented PUs increased with an increase in the hard segment content due to the rise of more continuous hard phase morphology. Figure 4 shows the ambient stress–strain behavior of the four segmented PUs, which were uniaxially deformed until failure [84].

### 6.3. Thermal Properties of Nanofiller–TPU Nanocomposites

The thermal stability of the TPU matrix is of great importance since its degradation commences around 230–300 °C. It is important to note that a TPU matrix possesses two glass transition temperatures (*T_g_*) corresponding to the soft and the hard segments, while it is known that the hard segment temperature *T_g_* is not always observed due to the dominance of the soft segment and ordered hard domains [85]. It also appears that the addition of a small amount of tethered clay could increase *T_g_* of the hard segment by 44 °C [52] while a 13 °C rise was observed in the *T_g_* value of the soft segment with the addition of Cloisite^®^ 20A [86]. TPU was found to exhibit two stages of degradation during decomposition. The first stage of degradation relates to the degradation of the hard segment and the second stage of degradation relates to the degradation of the soft segment [87]. In some other studies, a lower *T_g_* of the nanocomposite, as compared to the TPU matrix, has been reported [88]. The thermal stability of the clay–TPU nanocomposite will increase after the complete decomposition of the modifier compared to that of the TPU matrix [89]. An increase in the amount of clay from 1 to 5 wt.% was reported to increase the thermal stability of the TPU matrix from 12 to 34 °C, respectively [52]. The increase in thermal stability with an increase for MMT in the TPU matrix has also been reported in previous studies [56]. The improvement in thermal stability of the clay–TPU nanocomposite was found to be directly connected to the degree of dispersion of the nanoclay in the TPU matrix [89]. Enhancement of the thermal stability, along with the flame-retardant property of the clay–TPU nanocomposite, has been observed [90]. The thermal stability was enhanced by 25 °C with 5 wt.% clay. The heat release rate (HRR) decreased by 63% with the addition of 6% clay as compared to that of the TPU matrix. However, HRR decreased with further increase in the clay content. The degradation of the modified wt.% of carbon nanofiber (CNF) loading (PR-24-HHT-XT-LD) shows that the first- and second-onset degradation temperatures (Td1 and Td2) are significantly increased. It was also shown that the 50% weight loss degradation temperature (T50 wt.%) was positively shifted from about 400 °C for TPU matrix to 425 °C in a case of CNF-reinforced materials.

**Figure 5:**
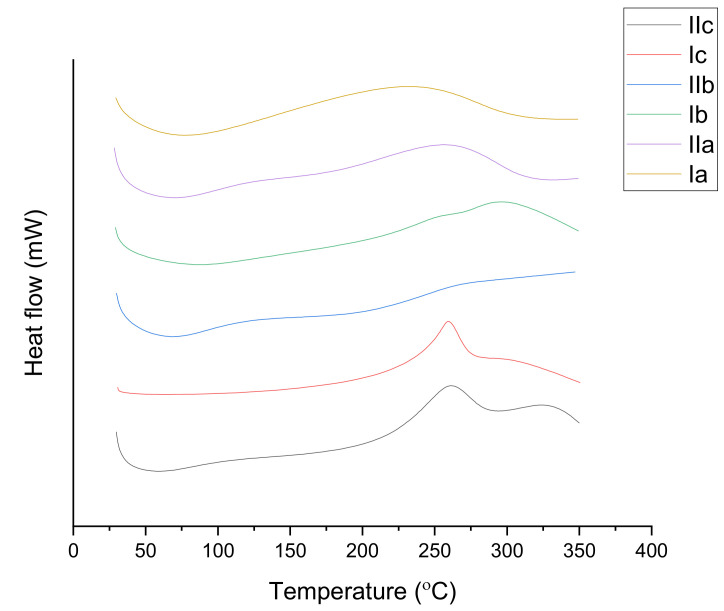
Differential scanning calorimetry (DSC) thermograms of PUs at a heating rate of 10 °C/min under a nitrogen atmosphere [15].

Kariduraganavar et al. [15] demonstrated that cross-linked PUs with azobenzene chromophores show improved thermal stability. Besides that, it was discovered that no *T_g_* could be seen for the PUs that had thiadiazole chromophores as part of their composition before they decompose, which infers that the *T_g_* was above the decomposition temperatures (*T_d_*). In order to investigate this stability, they controlled the temporal and thermal stability of the second harmonic generation signs for the PUs. This data obtained as operations of the time were their findings, as presented in a straightforward and simplified manner in Table 4. The differential scanning calorimetry (DSC) and thermal gravimetric analysis (TGA) thermograms are presented in Figure 5 [15].

**Figure 6:**
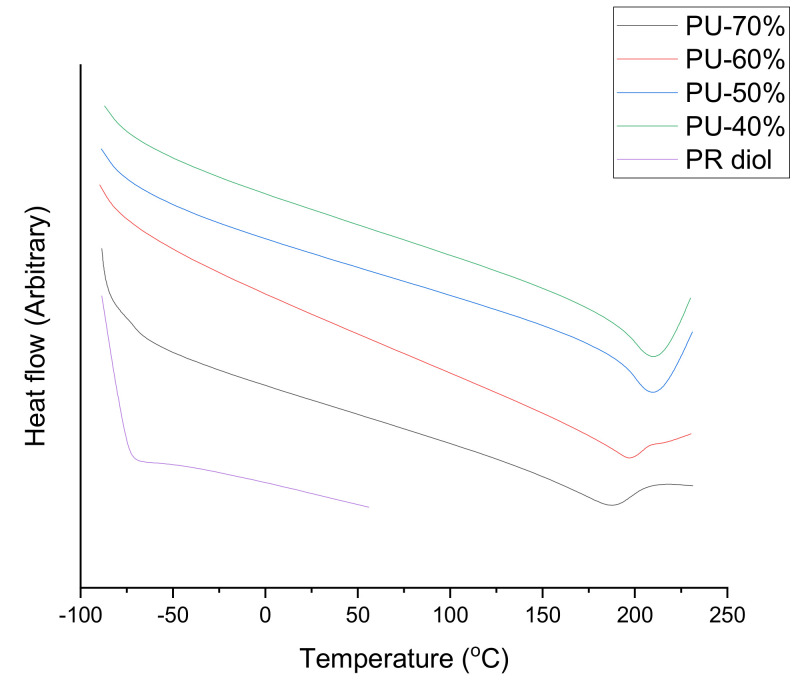
DSC thermograms of PUs at a heating rate of 10 °C/min [84].

Longer durations of soil exposure and vermiculite media produce harmful effects on the PU thermomechanical features and the composite, with a fall in the *T_g_* and in the storage modulus. In addition, a move towards higher temperatures for the initial thermal degradation occurred in the TGA curves of the composite and the neat PU linked to the lower concentration of dangling chains in the materials’ content of the exposed degrading media, as shown in Figure 6 [91].

Temperature operations show the PU effects on gas selectivity along with the penetrability of membranes. Previous research has centred on the modified chemical PU structure membrane to enhance its gas transport features [92]. Efforts to enhance the PUs’ thermal stability have been undertaken for a long period of time. One technique utilized to improve PU’s heat resistance is that of the structural chemical change via the introduction of thermally stable parts [93]. The flexible PU enables predictions to be used even for lower temperatures (assuming that this linear extrapolation was reliable for the entire temperature range) [94]. The thermal features, microstructure, mesomorphic features, and thermal degradation were examined for the PUs [95]. Findings from a dynamic contact angle showed enhanced hydrophobicity of the PU acrylic dispersion films [96]. The endotherm of this fluorinated phosphatidylcholine PUs was not seen at higher temperature areas in DSC curves, which are shown in Figure 6.

Based on the thermogravimetric experiments, PUs that were made with the use of a catalyst in nitrogen were more stable [97]. This cured hybrid showed higher thermal stability and more favourable mechanical strength as compared to pure PU [76]. In terms of PU, for many polymers, it is used as a flame-retardant new additive [98,99]. The use of the polyimide composition in PU improved the decaying temperature of PU for higher temperatures [100]. Czech et al. [100] put forward the finding that PU’s increase of length by raising the macrodiol length or by increasing the quantity of repeating units enabled the polymer to be softer at high temperatures. The reported *T_g_* of the systems derived by DSC are listed in Table 5, together with the definition of the samples’ nomenclature [100].

It is noted that minicolumns in flow systems, which are filled with loaded PU foam, did not depict any overpressure or swelling, although this often occurs with the utilization of other sorbents [101]. Madru et al. [102] had the contention that the water swelling of PU membranes was related to both its aggregation condition, as well as the temperature [102].

### 6.4. Electrical Properties of Nanofiller–TPU Nanocomposites

The electrical properties of a material are characterized by the ability of the material to conduct or insulate electricity based on the parameters such as its resistivity, conductivity, dielectric strength, temperature coefficient of resistance, and thermoelectricity [103]. Conductive fibres are known to improve electrical conductivity in polymer composites [104]. Examples of these are carbon, silver, gold, copper, and nickel, which are found in many different shapes and sizes [104]. In the composite field, the maximum electrical conductivity of 10 S/m at a loading of 7.4 wt.% of MWCNT and 0.3 wt.% as the percolation threshold for MWCNT/polyimide (MWCNT/PI) composite was achieved using the in situ polymerization method based on a study by Jiang et al. [105]. This concluded the ability of CNT to improve the EC of a polymer composite [106].

In the study of Vaithylingam et al. [107], the EC value of 0.33 S/m was noted to have been achieved at 7 wt.% loadings of MWCNT, and 5 wt.% was the percolation threshold for MWCNT/PU composite using the three-roll milling machine technique. It was determined that this technique is fast, simple, and easy, and compatible with standard industrial techniques enabling the production of high percolation threshold composite compared to in situ polymerization, solution compound, and combination of different techniques [107]. The addition of CNT can result in high dielectric strength at a very low loading fraction (<5 wt.%) being achieved. CNT adding may also change the electrical resistance of the composite under applied load/strain. This is called piezoresistivity and can be measured using a two-probe method. The composite with 5 wt.% loadings of MWCNT showed 92.92%, piezoresistivity, the largest percentage achieved [107]. Further loading of CNT into the composite may actually decrease the EC, piezoresistivity, and dielectric strength because of entanglement and agglomeration [107]. Based on Fu et al. [108], the degree of agglomeration of the 1-dimensional CNT can be reduced using two-dimensional graphite oxide (GO) as a novel dispersant to promote the adhesion between the CNT and PU matrix. The discovery of single-layer graphite, which is commonly known as graphene, has brought great improvements in the electrical properties of polymer composites [109].

In order to obtain high-yield graphene, top-down methods such as the combination of oxidation, exfoliation, and reduction of graphite are often practiced. GO is produced from exfoliation and oxidation of graphite. GO consists of a great number of versatile oxygen functional groups on its edge and basal planes [110]. This leads to stable dispersion in an aqueous solution [110]. Nevertheless, the disruption of the sp2 carbon structure by the oxygen functional groups results in GO with poor electrical conductivity. Chemical reduction is an effective method of obtaining reduced graphene oxide (RGO) which can be dispersed in aqueous or solvent media and solve the disruption of sp2 carbon structure [111]. Nevertheless, GO tends to aggregate and re-stack due to large van der Waals forces as well as π–π interactions [112]. Compatibilization of graphene via non-covalent and/or covalent interactions is an effective way to enhance surface adhesion with the polymer matrix and prevent aggregation. The existence of functional groups such as hydroxyl, carbonyl, and carboxyl promotes the covalent interaction. Hydroxyl groups can react with silicone coupling agents to result in better graphene adhesion to the polymer matrix [113]. Under the investigation of incorporating modified expanded graphene (EG), the electrical conductivity of 10^−1^ S/m at 8 wt.% loadings and 5.7 wt.% was taken as the percolation threshold for EG/PU composite using the in situ polymerization method [107].

In recent years, sulfonated graphite oxide (SGO) has been found to be of higher efficacy in facilitating the oxygen reduction reaction of GO. Hydroiodic acid (HI) may be used as a strong reducing agent since it is an environmentally friendly inorganic acid. The strength, as well as conductivity of GO and filled polymers increases after reduction with HI because of the reaction with the epoxy groups in GO. Hence, the reduced SGO (RSGO) has very good potential to improve the electrical and thermal conductivity of polymer composites [114]. Similar to CNT, introducing graphene to the polymer matrices improves the EC, piezoresistivity, and dielectric strength of the composite by a substantial degree [115].

In the past few years, silver nanoparticles (AgNP) have drawn much attention as it is widely used as conductive fibers for electrically conductive composites. *A_g_* is known for its high aspect ratio which is beneficial to form electrically conductive networks while having a relatively low percolation threshold in polymer composite. Nonetheless, *A_g_* has a tendency to agglomerate because of its fine size and large specific surface area, which could result in a reduction of the conductive path. *A_g_* is also prone to sedimentation as the density of *A_g_* (10.53 g/cm^3^) is higher than the polymer density (around 1.0 g/cm^3^). Therefore, is it required to address the *A_g_* sedimentation and agglomeration issue before proceeding with the preparation of Ag/polymer composite with high EC. The usage of Nanosilica (SiO_2_) as a dispersant may not be an option in this case because SiO_2_ is more of an electrical insulator. As a step to improve the performance of Ag/PU composite, GO or LDH may be used as a novel dispersant to improve the EC of said composite [107].

It was shown by Vaithylingam et al. [107] that the Ag/GO/PU composite exhibits superior electrical property (<10 Ω/sq) [116]. As global warming issues are on the rise, employing piezoelectric generator composites is able to convert vibration and mechanical energy source from human activities such as pressure, bending, and stretching motion into electrical energy. This is becoming a topic of high interest in recent composite studies. Material like zinc oxide (ZnO) and barium titanate (BaTiO_3_) can be used as fibers to improve the piezoelectric properties of the polymer composite. Based on the investigation by Vaithylingam et al. [107], the generator composite prepared using ZnO incorporated in the PU matrix showed the greatest peak voltage value of 40.45 V upon cyclic loading.

### 6.5. Chemical Properties of Nanofiller–TPU Nanocomposites

Chemical properties are conventionally used to determine the ability to resist or comply in a chemical reaction based on the parameters like toxicity, corrosion-resistance, chemical stability, flammability, and enthalpy of formation that governs composite classification [107]. The behaviour of the materials varies in terms of their resistance to corrosion such as monolithic materials. Prevention of corrosion can be performed by organic-based coatings. However, these materials may fail due to electrolyte exposure which is responsible for cathodic delamination and physical damage like impact, scratching, or wear during service. This allows direct contact of underlying steel substrates to aggressive environment and electrochemical reaction taking place at the coating–substrate interface [117]. The increase in hydroxide ions concentration results in alkalization weakening the adhesion of steel substrate and coating. In addition, it was found that transporting water and oxygen molecules through organic coatings could be critical in reducing oxygen [118].

Recent research has shown that adding nanoparticles (NP) such as SiO_2_, TiO_2_, ZnO, Al_2_O_3_, MWCNT, and CaCO_3_ in coatings results in improving the thermal stability of the polymer and enhancing scratch and abrasion resistance [119]. As an example, incorporating MWCNT in polymer matrices improves the wear resistance and reduces the friction of the composite when upgraded [120]. It was reported that increasing MWCNT content from 0 to 0.5 wt.% causes decreasing of the PU cathodic delamination which is caused by making a dense barrier of MWCNT, which, in turn, blocks the oxygen and water molecule pathways through the coating [58].

### 6.6. Acoustic Properties of Nanofiller–TPU Nanocomposites

In the acoustic behaviour of materials, the cellular structure can be used to absorb sound [121]. The determination of the acoustic properties of a material depends on the ability to perform as either insulator or conductor by involving vibration, ultrasound, and infrasound [122]. Examples of acoustic materials include nanoclay, titania nanoparticles (TiO_2_), and MWCNT which can be incorporated into the PU matrix to enhance sound absorption [93,123]. It has been reported that the MWCNT/SiO_2_/PU composite showed remarkable sound insulation properties compared to pristine PU where the addition of 0.2 wt.% SiO_2_ and 0.35 wt.% CNT to the PU composition improved sound transmission loss up to 80 dB that of pure PU foam sample [124]. Surprisingly, the continuous addition of CNT of up to 2 wt.% had poorer sound insulation than the pristine PU foam due to the high loading of CNT effectively preventing the interactions between the polyol and isocyanate [125].

### 6.7. Viscoelastic Properties of Nanofiller–TPU Nanocomposites

Viscoelastic properties of a composite refer to the ability to exhibit both viscous and elastic characteristics when undergoing deformation [126]. These materials are resisting the shear flow and strain linearly upon stretching; yet, they can retain their shapes as the stress is removed [127].

Research in this field has led to the discovery of smart materials such as electro active polymers (EAP).

EAPs have the ability to change their size or shape when stimulated by the right external electrical activation mechanism by converting the electric signal into energy [128]. PU-based composites are very favourable when taking viscoelastic properties into consideration as they are flexible, lightweight, biocompatible, easy to process, and have the ability to be moulded into various shapes [129]. The authors investigated incorporating grafted CNT into the PU matrix using the “grafting onto” technique to increase the interfacial adhesion, dielectric strength, permittivity, and 5 wt.% as the percolation threshold of the CNT/PU composite. Further, the electromechanical performance of the composite was also increased by a factor of 2, thus enabling high viscoelastic behaviour.

## 7. Applications

**Figure 7:**
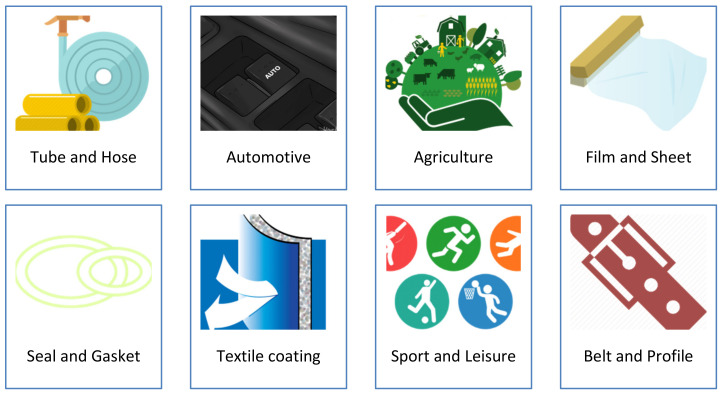
Applications of TPU [132].

TPU fortified by its exceptional mechanical properties and biocompatibility is widely considered for the construction of implantable medical components by replacing silicon for implantation [44,130]. The reason for this potential replacement is mainly due to TPU’s superior tensile and tear strength which allows thinner insulation and more intricate design, while still maintaining structural integrity [131]. Figure 7 shows the general applications of TPU.

### 7.1. Additive Manufacturing (AM)

AM is layer-by-layer 3D object printing. AM is mainly related to using polymers and their composites in modern industries and it has been proven that AM has vast potential for various applications, especially in the medical, aerospace, and automotive industries. AM techniques have been developing due to the creation of new materials and techniques such as photo polymerization, material jetting, powder bed fusion, material extrusion, binder jetting, and sheet lamination. The focus of most research in the AM field is to investigate the possibility to conduct a specific application, process, or type of material. The first step in this search is to highlight the progress of the corresponding materials and the methodology of the preparation of these materials. However, there are many challenges in using AM technology for polymer materials [54].

### 7.2. PU in Human Soft Tissues

**Figure 8:**
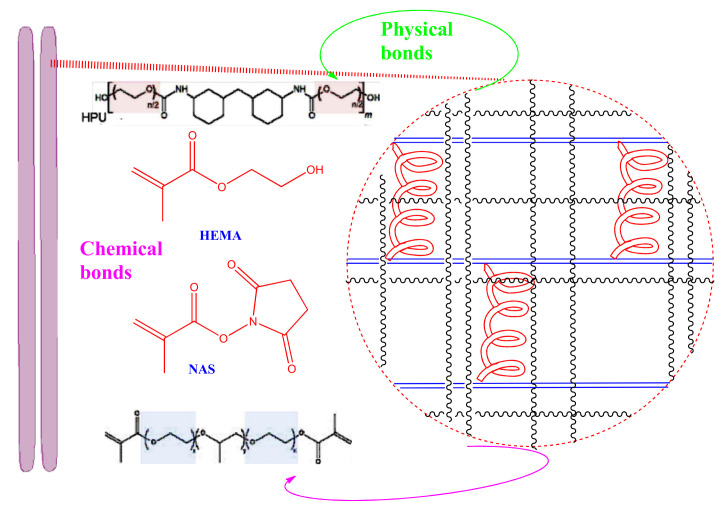
Hydrogel PU used in soft human tissues [136].

The PU composite has a greater amount of water (over long timescales) when compared to both media due to the extremely hygroscopic properties of wood flour particles, commonly known as sawdust [133]. One of the applications of TPU could be possibly related to biocompatible tough hydrogels in medical devices, such as prosthetics. The important key that causes the hard and soft segments located in the topography of the PU parts is what determines the strength of the hydrogen bonds [134]. In addition, the average particle size can be changed or modified by varying the emulsification procedures, such as the revolution speed of the mixer or temperature in PU dispersions. Furthermore, water absorption into the particles showed an increase in concurrence with the increasing hydrophilicity of the PU [135]. There is a series of biocompatible tough hydrogels whose mechanical properties can be adjusted by fabricating a physically crosslinked poly(ethylene glycol)-based polyurethane and a copolymer. There is a significant effect of varying the chain length of the chemical crosslinkers and the chain length of the hydrophilic soft segments in the physically crosslinked polyurethane. This effect is linked to the physicochemical properties of the hydrogels which are widely used in determining the swelling, stiffness, strength, and toughness. The manipulation of the length of polyethylene glycol results in changing the length of polyethylene oxide; the networks become tightened or loosened, as depicted in Figure 8 [136].

### 7.3. Coating and Anti-Corrosion and Anti-Bacterial Properties

**Figure 9:**
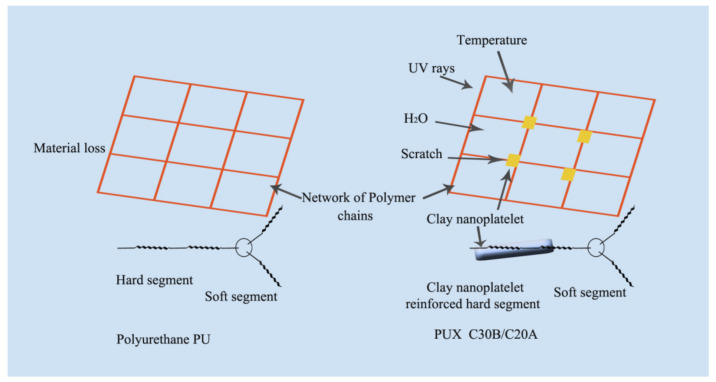
Atomic Force Microscopy (AFM) investigation of modified PU morphology [137].

Another application is the use of the modified polyurethane (PU) in Figure 9 which was prepared by using polyol with toluene diisocyanate in NCO/OH = 0.7 ratios. Investigation of the prepared polymer by spectroscopic studies has confirmed the modification process. The anti-corrosive properties of the galvanized steel with PU coating surpassed the steel in an aqueous (3.5 wt%) NaCl solution environment. Atomic Force Microscopy (AFM) showed the surface hydrophobicity, roughness, and morphology. The moity cross-link in the PU backbone chain has expressed better physicochemical properties compared to the unmodified PU. In addition, the thermal stability of modified coated PU was investigated by differential scanning calorimetry (DSC) and thermal gravimetric analysis (TGA). The results have shown better surface qualities in terms of corrosion [137].

### 7.4. PU Foam Uses

The weight loss of PUs decreases with increasing spacer lengths. Asensio et al. [42] showed that the patterns in flexible PU foams correlate with their plaque which provides the rationale for the utilization of plaques. The radiolabel that is released from incubations is normalized based on the radioactivity of the PU used [87]. The depolymerized oligoesters could be esterified with coco fatty acid so that a polymeric precursor, polyester polyol for PU can be built [138]. Wang et al. [139] mentioned the need and importance of hydrogen linking and inductive elements in determining the phase of PUs. The structure of PUs is depicted in the insert of Table 1. A wide variety of elements can be achieved by tuning the structure of the PU [140]. It was concluded that nitrogen plasma was the correct and best approach for improving the wetting properties of a PU film [141]. This was supported by Datta and Kasprzyk [142] who showed that polyricinoleate-segmented PU was stable enough to be processed using injection molding and extrusion [142]. This is of concept if traditional stabilizers were additions, as is the practice in the industry [143]. According to another study [144], the incorporation of PETpc particles into the PU foam resulted in a reduction in the mass loss for both top and bottom layers while cycling [144].

Both elastomers, as well as thermal degradation of PU foams, have been extensively studied in the last half a century, with differences and inconsistencies in results and findings because of a variety of PU compositions, materials, and products. Isocyanate, polyol, and chain extender have been used to produce the PUs, with the different linkages in the polymer chain resulting in different thermal dissociation temperatures [145]. Early degradation temperature in hard segments for PUs has been successfully reduced through the decreased composition of the soft segment [146,147]. Furthermore, the thermal degradation of PUs was examined thoroughly for the different types of diisocyanate (aliphatic or aromatic), polyol (polyester or polyether), and chain extender, where diisocyanates may be aliphatic or aromatic and polyols may be polyesters or polyethers.

### 7.5. Environmental Applications

The degradation system was also seen to be very complex due to the variety of products in the process. However, this seems to be the case only at the beginning stage of degradation which may be due to the high quantity of aromaticity in the polyester backbone causing the PU chains to be susceptible to scission, and this solved challenges of the structure crowing. The degradation of the PU films may not be a result of the polymer backbone’s breakage but may instead be due to reduced cohesive energy within the hydrazo PU chains [148]. Ourique et al. [149] stated that the degradation kinetics were slower for the structured material when compared to the supple PU. The very slow degradation kinetics that was measured shows that the PUs fit long-term underwater applications [149,150]. It was clear that the other PUs would continuously degrade following testing, beyond the test period. PUs with these isocyanates showed unfavourable mechanical features when compared to PUs with traditional isocyanates and hydrolytic degradation of PUs that were slow [151].

The thermal degradation of PU was noted by the decomposition of urethane links, the degradation of soft segments, and the volatile components’ evolution [152]. The same authors have mentioned that this was not due to only pure thermal oxidation but rather also to prior degradation of the PU sample. Degradation of PUs may cause unfavourable issues by hydrolytic reactions which are of importance in polyester-based PUs [153]. Khadivi et al. [154] maintained that the degradation products via the PUs and nanocomposites showed no cytotoxic effect. Furthermore, steady differences in cytotoxicity of the by-products from polymers were not seen. Moreover, it was predicted that the polymers would be viable for ophthalmological uses. In simulated physiological situations, PU samples showed a more substantial weight loss when in vitro degradation assessments were undertaken [155]. The polyether with PUs had no significant signs of cell-mediated degradation under the same conditions as determined by radiolabel release. A live cell culture system used to test the susceptibility of PUs to degradation was an important measure in studying and understanding the mechanism of biodegradation [87]. The findings depict that the nanocomposites degradation rates turned slightly slower as compared to PU, implying PU’s improvement of thermal stability, as the inorganic material could be a barrier for the heat to expand fast and limit further degradation [156].

## 8. Conclusions

The chemical structure and morphological annealing of PU have been reviewed experimentally and theoretically to investigate the phase-separation kinetics in terms of the influence of the powerful hydrogen link between the hard and soft segments of urethane. Controlling the interaction between hard and soft segments through hydrogen linking plays a crucial role in featuring the amorphous structure of PU and the thermally labile thermoplastic polyurethane (TPU), which is chemically similar to PU with a better heat resistivity. TPU has become the most versatile engineering thermoplastic with exceptional mechanical properties and biocompatibility that could be utilized to develop more interesting polymers due to specific structures of TPU macromolecules, interphase interactions, and microphase transformations. In addition, polydimethylsiloxane (PDMS)-based PUs were investigated and it was found that it is well-equipped with bio stability caused by the mixed macrodiol technique.

TPU and PU were investigated commercially and technically to highlight the incorporation of inorganic nanofillers such as carbon or clay using a process. The most important step is to incorporate TPUs in many possible applications relying on biocompatibility properties. Meanwhile, exfoliated morphology occurred when a 40 wt. % nanoclay was used to utilize a pseudo chain extender. The resulting PU-nanocomposite of 5 wt.% Cloisite^®^30B has shown an increase in modulus and tensile strength by 110 and 160%, respectively, compared to the 30–90% increase of the nanocomposite PU–0.5 wt.% CNTs. Adversely, the high decrease in the strength was attributed to the rise in PU foam content, which in turn was a result of an increase in mass loss. Coating PU was found to influence stress–strain behaviour due to the interaction between the soft segment and physical crosslinkers.

The thermophysical properties of the TPU matrix have shown two glass transition temperatures (*T_g_*’s) corresponding to the soft and the hard segment. *T_g_* for both segments is influenced by adding a small amount of tethered clay shifting temperature for hard and soft segments by 44 °C and 13 °C, respectively. Meanwhile, an increase of clay content from 1 to 5 wt.% results in increasing the thermal stability of the TPU matrix from 12 to 34 °C, respectively. The shifting temperature of TPU–CNF nanocomposites from 400 °C to 425 °C could be caused by experiencing 50% weight loss. The phase structure of PU dispersion investigated by DSC suggested enhancing properties such as thermal stability, solubility, and flexibility. The maximum piezoresistivity measured by the electrical conductivity of PU composites of 7.4 wt.% MWCNT could hit 10 S/m, reaching 92.92%.

The chemical structure of the PU–CNT composite has shown a degree of agglomeration which, under disruption of the sp2 carbon structure, shows poor electrical conductivity. However, with expanded graphene loading at 5.7 wt.%, piezoresistivity could hit 10^−1^ S/m, less than 100 times than PU. Another composite, MWCNT (0.35 wt.%)/SiO_2_ (0.2 wt.%)/PU, has shown excellent sound absorption of 80 dB compared to the PU foam sample. Recently, adding nanoparticles such as SiO_2_, TiO_2_, ZnO, Al_2_O_3_ results in improving the thermal stability of the polymer and enhancing scratch and abrasion resistance.

## Figures and Tables

**Table 1:** Comparison property between thermoset polyurethane (PU) and thermoplastic polyurethane (TPU) (partially from Kopal et al. [48]).

Thermoset PU	Thermoplastic PU
Less hardness	High hardness
Burns easily	Difficult to burn
Soft and delicate	Rough, strong
Moderate abrasion resistance	High abrasion resistance
Withstands temperatures up to 250 °C	Softens and deforms above 250 °C
Hold a large amount of weight	Unable hold a large amount of weight
Specific gravity: 1 to 1.2	Specific gravity: 1.2
Shore hardness (HV): 10–85	Shore hardness (HV): 55
Tensile strength (MPa): 1–12	Tensile strength (MPa): 48–83
Elongation at break (%): 10–510	Elongation at break (%): 500
Tear strength (N/m): 6–48	Tear strength (N/m): 200
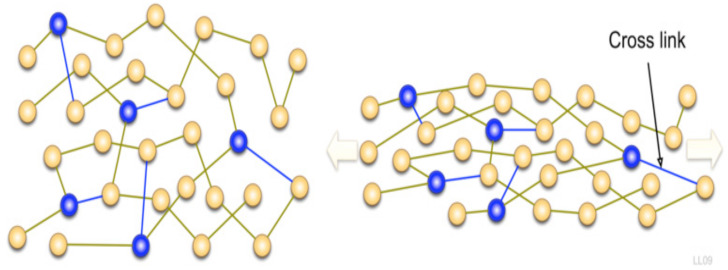	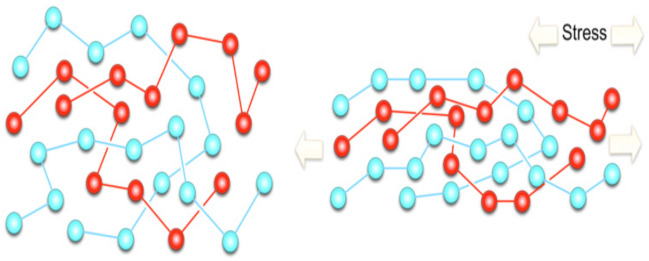

**Table 2:** Basic and valuable properties of TPU (Partially from Tehran et al. [49]).

Property	Value
Density	1224 kg/m^3^
Shore hardness	55 A
Tensile strength	20 MPa
Melting temperature	200 °C
Ultimate elongation	500 %
Glass transition temperature	−42 °C
Low temperature brittle point	≤−68 °C
Injection molding–melt temperature	200–220 °C
Injection molding–mold temperature	20–40 °C
Maximum drying temperature	110 °C

**Table 3:** Mechanical properties of PUs with different hard segment contents [75].

Type of PU	Rm (MPa)	E (MPa)	ε (%)	Hardness (°Sh D)
PU2PCL2000	>24 ± 1	39 ± 1	>763 ± 41	41 ± 2
PU2PCL1250	23 ± 2	17 ± 3	643 ± 40	22 ± 1
PU4PCL2000	33 ± 3	36 ± 2	440 ± 56	27 ± 2
PU4PCL1250	48 ± 3	39 ± 2	356 ± 11	38 ± 2
PU2PCL530	41 ± 2	38 ± 0.9	420 ± 28	43 ± 2
PU4PCL530	1.6 ± 0.4	32 ± 0.4	-	61 ± 3

**Table 4:** Physical properties of PUs [15].

PUs	*T_g_* (°C)	*T_opt_* (°C)	Field Applied (kV)	*T_d_* (°C)
Ia	140	140	2.0	207
IIa	147	145	3.0	221
Ib	155	150	3.0	227
IIb	165	170	3.0	273
Ic	-	180	2.5	250
IIc	-	205	3.0	276

**Table 5:** The *T_g_* determined from the DSC second scans for the PU-based networks crosslinked with hyperbranched polyester (Boltorn H40) [100].

PU Network Name	Number of Repeating Units in the PU Chain	Molecular Weight of Terathane (g/mol)	*T_g_* (°C)
20-T650	20	650	−43.5
20-T1000	20	1000	−59.5
20-T2000	20	2000	−77.3
20-T2900	20	2900	−82.1
04-T2000	4	2000	−77.7
10-T2000	10	2000	−77.0
